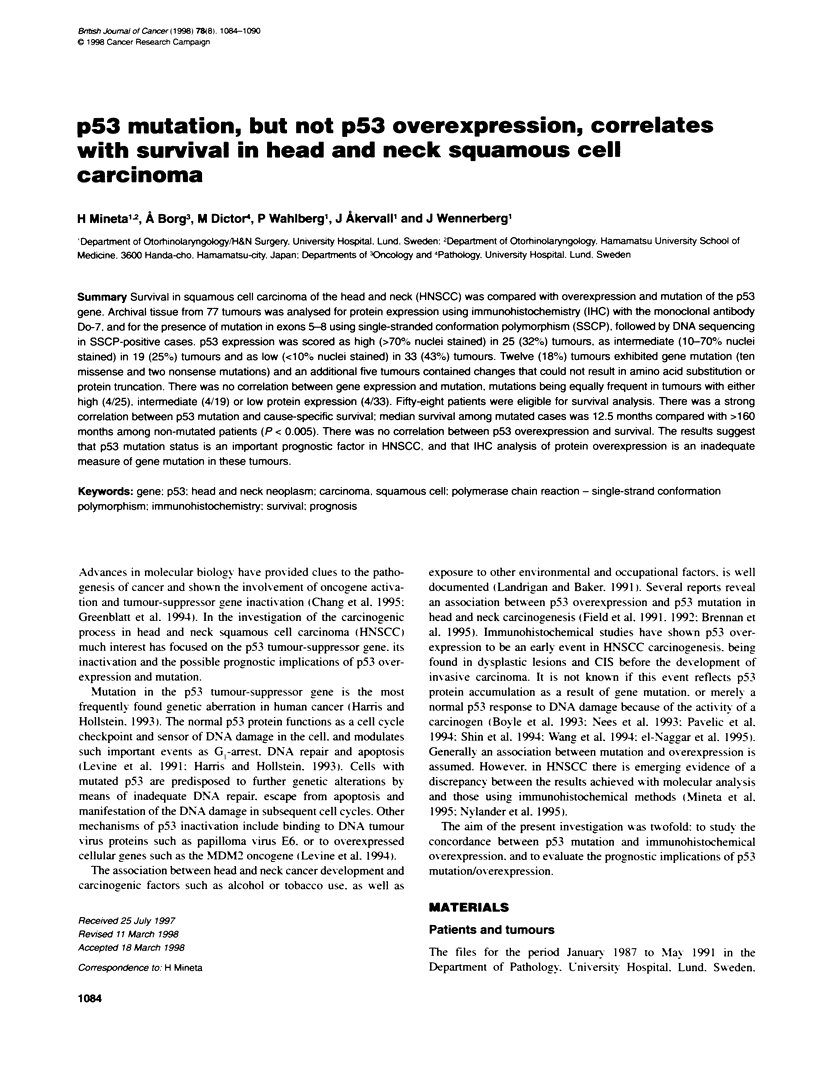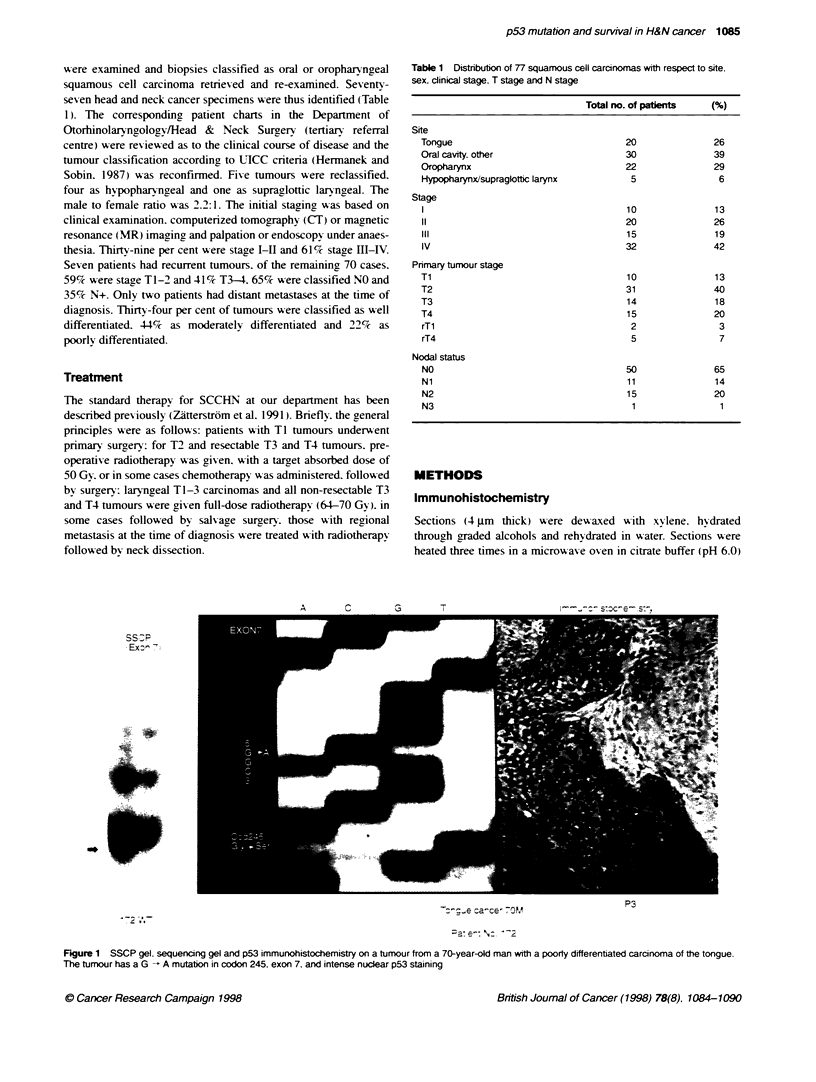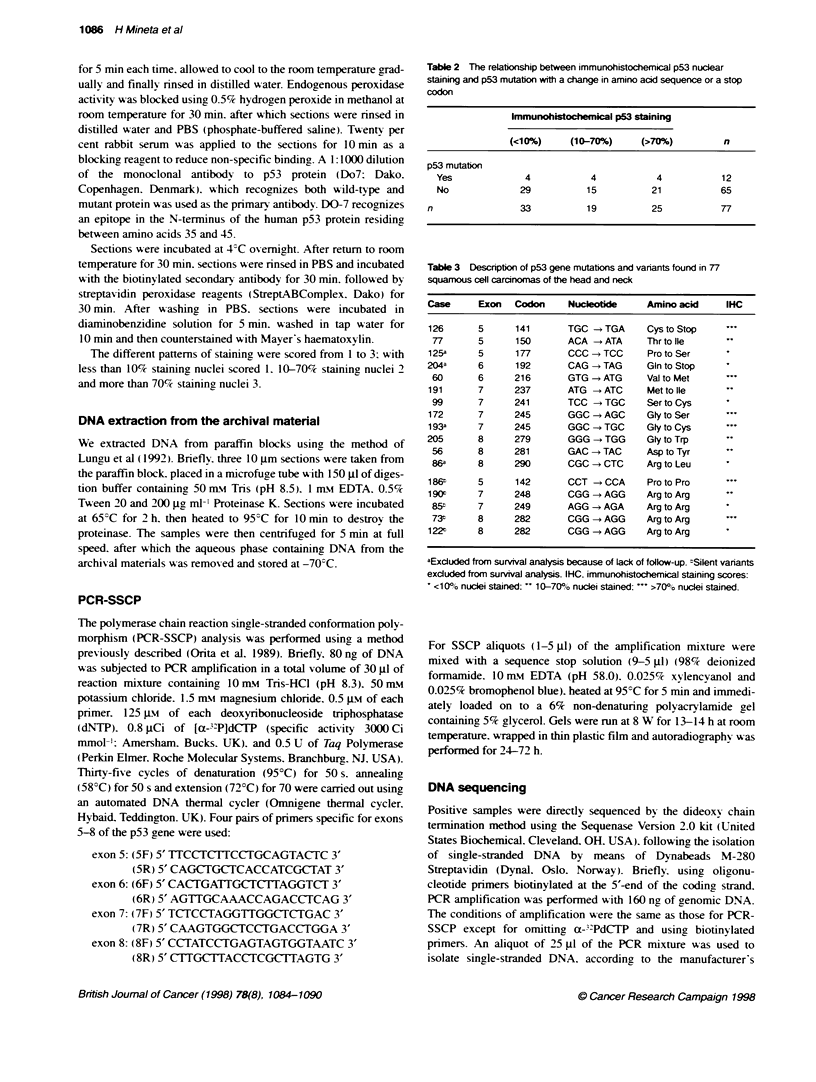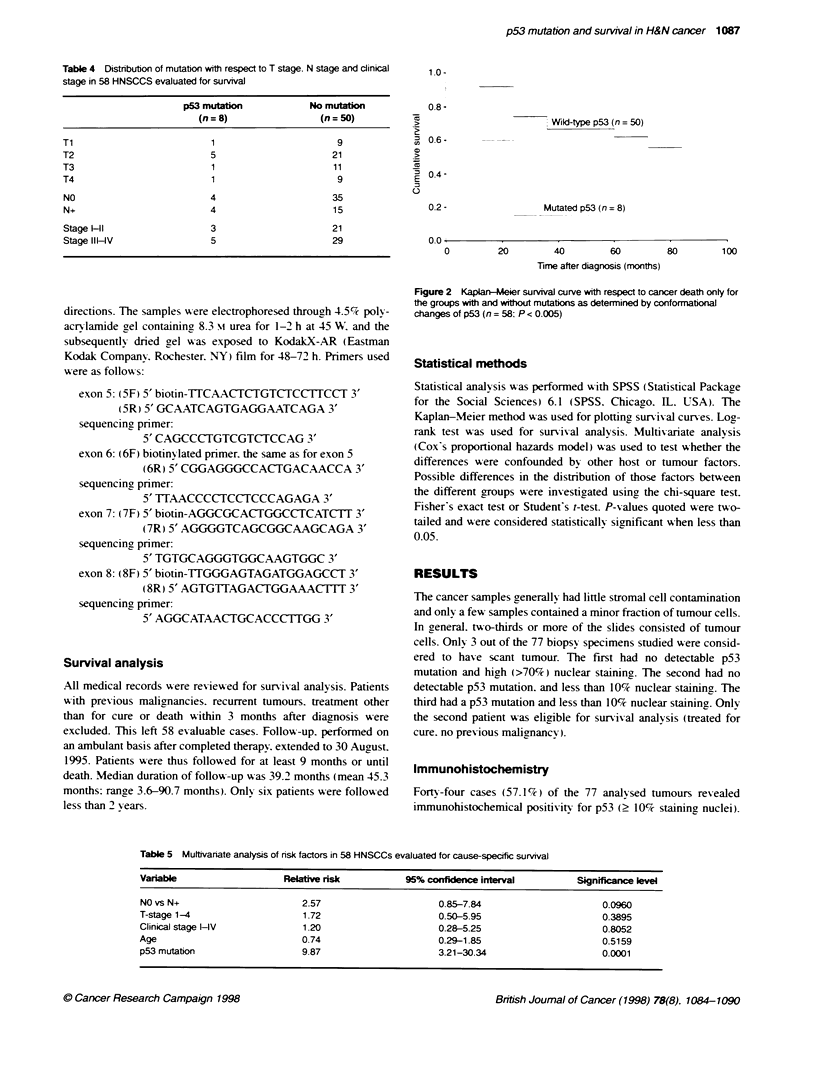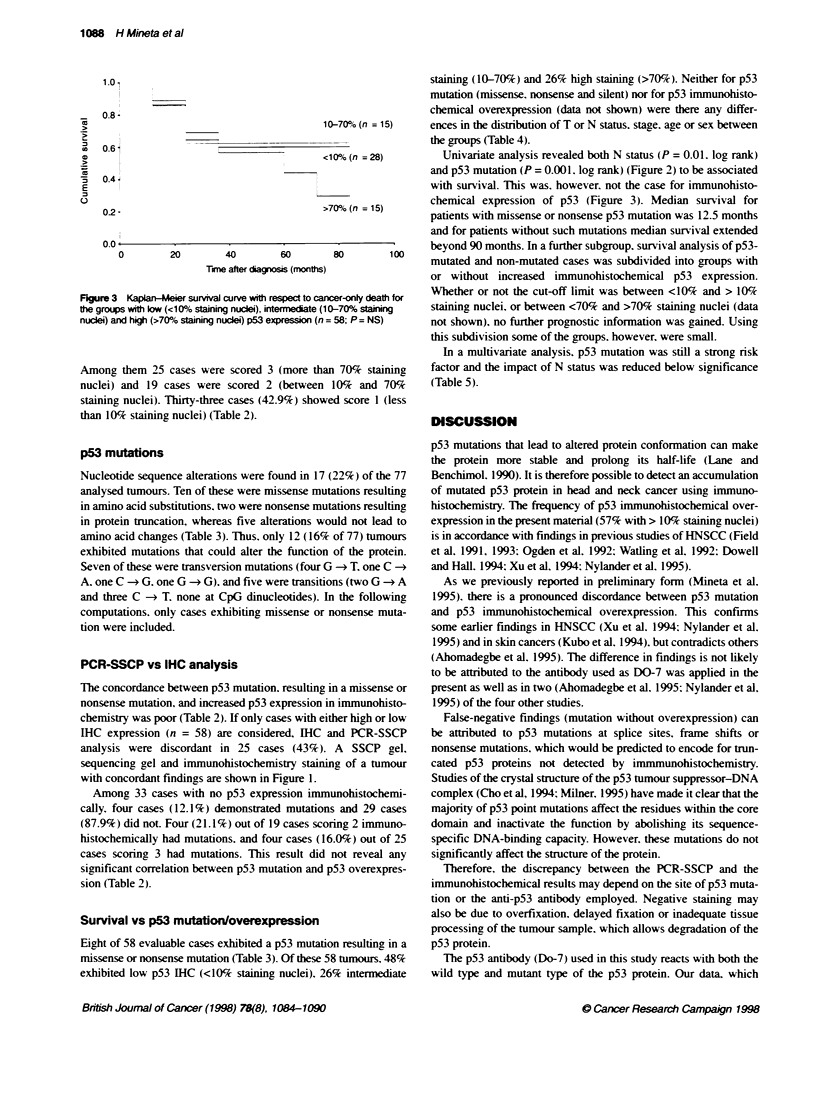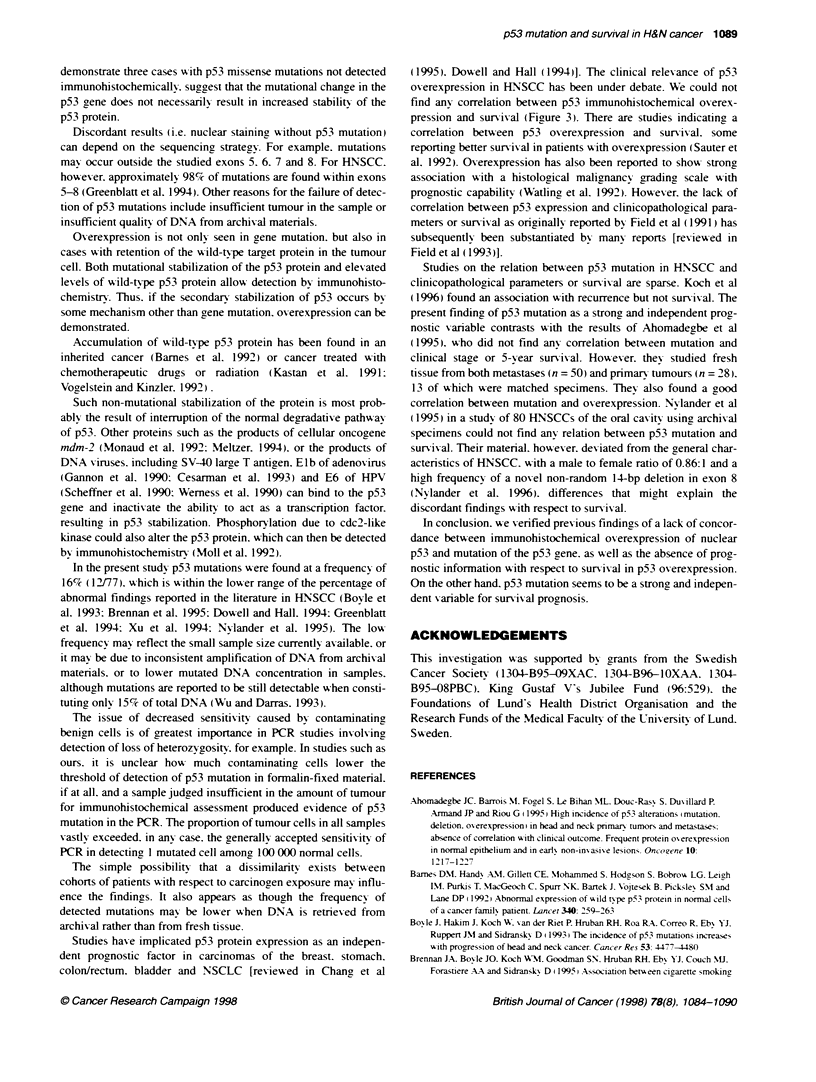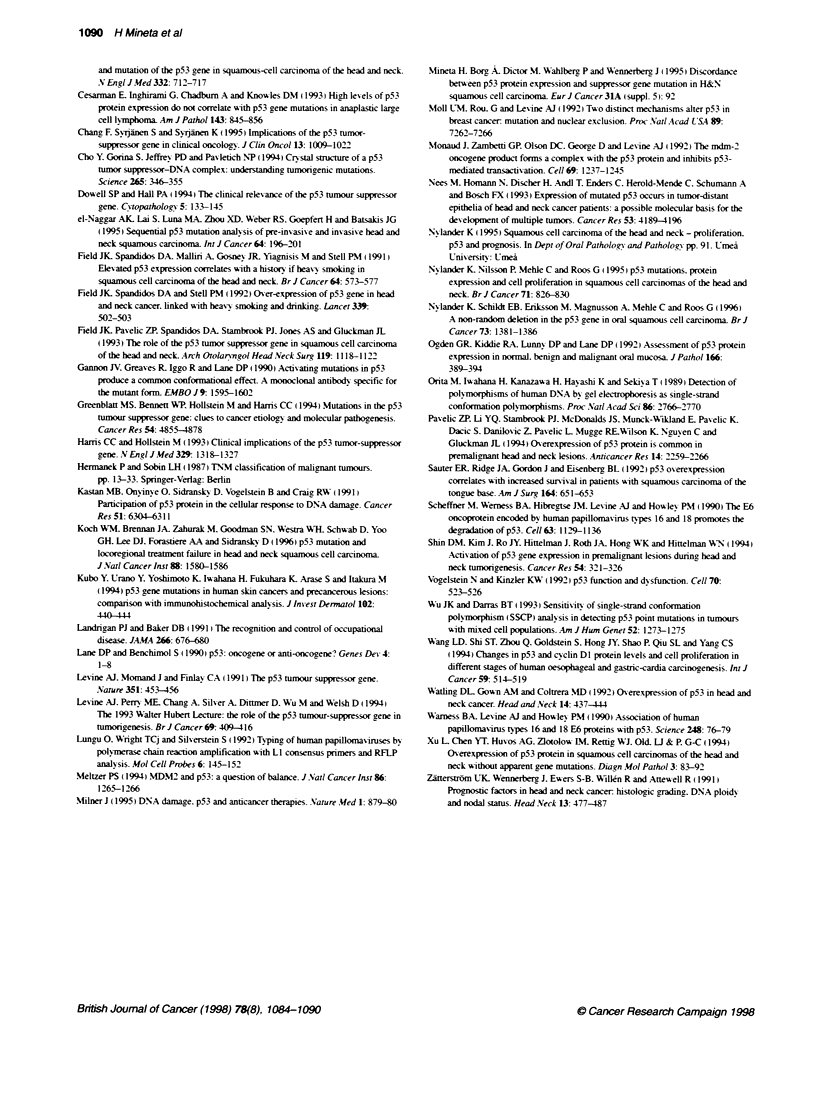# p53 mutation, but not p53 overexpression, correlates with survival in head and neck squamous cell carcinoma.

**DOI:** 10.1038/bjc.1998.632

**Published:** 1998-10

**Authors:** H. Mineta, A. Borg, M. Dictor, P. Wahlberg, J. Akervall, J. Wennerberg

**Affiliations:** Department of Otorhinolaryngology/H&N Surgery, University Hospital, Lund, Sweden.

## Abstract

**Images:**


British Journal of Cancer (1998) 78(8), 1084–1090
© 1998 Cancer Research Campaign

### p53 mutation, but not p53 overexpression, correlates with survival in head and neck squamous cell carcinoma

**H Mineta[1,2], Å Borg[3], M Dictor[4], P Wahlberg[1], J Åkervall[1] and J Wennerberg[1]**

[1]Department of Otorhinolaryngology/H&N Surgery, University Hospital, Lund, Sweden; [2]Department of Otorhinolaryngology, Hamamatsu University School of Medicine, 3600 Handa-cho, Hamamatsu-city, Japan; Departments of [3]Oncology and [4]Pathology, University Hospital, Lund, Sweden

**Summary** Survival in squamous cell carcinoma of the head and neck (HNSCC) was compared with overexpression and mutation of the p53 gene. Archival tissue from 77 tumours was analysed for protein expression using immunohistochemistry (IHC) with the monoclonal antibody Do-7, and for the presence of mutation in exons 5–8 using single-stranded conformation polymorphism (SSCP), followed by DNA sequencing in SSCP-positive cases. p53 expression was scored as high (>70% nuclei stained) in 25 (32%) tumours, as intermediate (10–70% nuclei stained) in 19 (25%) tumours and as low (<10% nuclei stained) in 33 (43%) tumours. Twelve (18%) tumours exhibited gene mutation (ten missense and two nonsense mutations) and an additional five tumours contained changes that could not result in amino acid substitution or protein truncation. There was no correlation between gene expression and mutation, mutations being equally frequent in tumours with either high (4/25), intermediate (4/19) or low protein expression (4/33). Fifty-eight patients were eligible for survival analysis. There was a strong correlation between p53 mutation and cause-specific survival; median survival among mutated cases was 12.5 months compared with >160 months among non-mutated patients ($P < 0.005$). There was no correlation between p53 overexpression and survival. The results suggest that p53 mutation status is an important prognostic factor in HNSCC, and that IHC analysis of protein overexpression is an inadequate measure of gene mutation in these tumours.

**Keywords:** gene; p53; head and neck neoplasm; carcinoma, squamous cell; polymerase chain reaction – single-strand conformation polymorphism; immunohistochemistry; survival; prognosis

Advances in molecular biology have provided clues to the pathogenesis of cancer and shown the involvement of oncogene activation and tumour-suppressor gene inactivation (Chang et al, 1995; Greenblatt et al, 1994). In the investigation of the carcinogenic process in head and neck squamous cell carcinoma (HNSCC) much interest has focused on the p53 tumour-suppressor gene, its inactivation and the possible prognostic implications of p53 overexpression and mutation.

Mutation in the p53 tumour-suppressor gene is the most frequently found genetic aberration in human cancer (Harris and Hollstein, 1993). The normal p53 protein functions as a cell cycle checkpoint and sensor of DNA damage in the cell, and modulates such important events as $G_1$-arrest, DNA repair and apoptosis (Levine et al, 1991; Harris and Hollstein, 1993). Cells with mutated p53 are predisposed to further genetic alterations by means of inadequate DNA repair, escape from apoptosis and manifestation of the DNA damage in subsequent cell cycles. Other mechanisms of p53 inactivation include binding to DNA tumour virus proteins such as papilloma virus E6, or to overexpressed cellular genes such as the MDM2 oncogene (Levine et al, 1994).

The association between head and neck cancer development and carcinogenic factors such as alcohol or tobacco use, as well as exposure to other environmental and occupational factors, is well documented (Landrigan and Baker, 1991). Several reports reveal an association between p53 overexpression and p53 mutation in head and neck carcinogenesis (Field et al, 1991, 1992; Brennan et al, 1995). Immunohistochemical studies have shown p53 overexpression to be an early event in HNSCC carcinogenesis, being found in dysplastic lesions and CIS before the development of invasive carcinoma. It is not known if this event reflects p53 protein accumulation as a result of gene mutation, or merely a normal p53 response to DNA damage because of the activity of a carcinogen (Boyle et al, 1993; Nees et al, 1993; Pavelic et al, 1994; Shin et al, 1994; Wang et al, 1994; el-Naggar et al, 1995). Generally an association between mutation and overexpression is assumed. However, in HNSCC there is emerging evidence of a discrepancy between the results achieved with molecular analysis and those using immunohistochemical methods (Mineta et al, 1995; Nylander et al, 1995).

The aim of the present investigation was twofold: to study the concordance between p53 mutation and immunohistochemical overexpression, and to evaluate the prognostic implications of p53 mutation/overexpression.

*Received 25 July 1997*
*Revised 11 March 1998*
*Accepted 18 March 1998*

*Correspondence to:* H Mineta

### MATERIALS

### Patients and tumours

The files for the period January 1987 to May 1991 in the Department of Pathology, University Hospital, Lund, Sweden, were examined and biopsies classified as oral or oropharyngeal squamous cell carcinoma retrieved and re-examined. Seventy-seven head and neck cancer specimens were thus identified (Table 1). The corresponding patient charts in the Department of Otorhinolaryngology/Head & Neck Surgery (tertiary referral centre) were reviewed as to the clinical course of disease and the tumour classification according to UICC criteria (Hermanek and Sobin, 1987) was reconfirmed. Five tumours were reclassified, four as hypopharyngeal and one as supraglottic laryngeal. The male to female ratio was 2.2:1. The initial staging was based on clinical examination, computerized tomography (CT) or magnetic resonance (MR) imaging and palpation or endoscopy under anaesthesia. Thirty-nine per cent were stage I–II and 61% stage III–IV. Seven patients had recurrent tumours, of the remaining 70 cases, 59% were stage T1–2 and 41% T3–4, 65% were classified N0 and 35% N+. Only two patients had distant metastases at the time of diagnosis. Thirty-four per cent of tumours were classified as well differentiated, 44% as moderately differentiated and 22% as poorly differentiated.

### Treatment

The standard therapy for SCCHN at our department has been described previously (Zätterström et al, 1991). Briefly, the general principles were as follows: patients with T1 tumours underwent primary surgery; for T2 and resectable T3 and T4 tumours, preoperative radiotherapy was given, with a target absorbed dose of 50 Gy, or in some cases chemotherapy was administered, followed by surgery; laryngeal T1–3 carcinomas and all non-resectable T3 and T4 tumours were given full-dose radiotherapy (64–70 Gy), in some cases followed by salvage surgery, those with regional metastasis at the time of diagnosis were treated with radiotherapy followed by neck dissection.

**Table 1** Distribution of 77 squamous cell carcinomas with respect to site, sex, clinical stage, T stage and N stage

| | Total no. of patients | (%) |
|---|---|---|
| Site | | |
| Tongue | 20 | 26 |
| Oral cavity, other | 30 | 39 |
| Oropharynx | 22 | 29 |
| Hypopharynx/supraglottic larynx | 5 | 6 |
| Stage | | |
| I | 10 | 13 |
| II | 20 | 26 |
| III | 15 | 19 |
| IV | 32 | 42 |
| Primary tumour stage | | |
| T1 | 10 | 13 |
| T2 | 31 | 40 |
| T3 | 14 | 18 |
| T4 | 15 | 20 |
| rT1 | 2 | 3 |
| rT4 | 5 | 7 |
| Nodal status | | |
| N0 | 50 | 65 |
| N1 | 11 | 14 |
| N2 | 15 | 20 |
| N3 | 1 | 1 |

### METHODS

### Immunohistochemistry

Sections (4 µm thick) were dewaxed with xylene, hydrated through graded alcohols and rehydrated in water. Sections were heated three times in a microwave oven in citrate buffer (pH 6.0)

**Figure 1** SSCP gel, sequencing gel and p53 immunohistochemistry on a tumour from a 70-year-old man with a poorly differentiated carcinoma of the tongue. The tumour has a G → A mutation in codon 245, exon 7, and intense nuclear p53 staining

© Cancer Research Campaign 1998

for 5 min each time, allowed to cool to the room temperature gradually and finally rinsed in distilled water. Endogenous peroxidase activity was blocked using 0.5% hydrogen peroxide in methanol at room temperature for 30 min, after which sections were rinsed in distilled water and PBS (phosphate-buffered saline). Twenty per cent rabbit serum was applied to the sections for 10 min as a blocking reagent to reduce non-specific binding. A 1:1000 dilution of the monoclonal antibody to p53 protein (Do7; Dako, Copenhagen, Denmark), which recognizes both wild-type and mutant protein was used as the primary antibody. DO-7 recognizes an epitope in the N-terminus of the human p53 protein residing between amino acids 35 and 45.

Sections were incubated at 4°C overnight. After return to room temperature for 30 min, sections were rinsed in PBS and incubated with the biotinylated secondary antibody for 30 min, followed by streptavidin peroxidase reagents (StreptABComplex, Dako) for 30 min. After washing in PBS, sections were incubated in diaminobenzidine solution for 5 min, washed in tap water for 10 min and then counterstained with Mayer's haematoxylin.

The different patterns of staining were scored from 1 to 3; with less than 10% staining nuclei scored 1, 10–70% staining nuclei 2 and more than 70% staining nuclei 3.

### DNA extraction from the archival material

We extracted DNA from paraffin blocks using the method of Lungu et al (1992). Briefly, three 10 µm sections were taken from the paraffin block, placed in a microfuge tube with 150 µl of digestion buffer containing 50 mM Tris (pH 8.5), 1 mM EDTA, 0.5% Tween 20 and 200 µg ml$^{-1}$ Proteinase K. Sections were incubated at 65°C for 2 h, then heated to 95°C for 10 min to destroy the proteinase. The samples were then centrifuged for 5 min at full speed, after which the aqueous phase containing DNA from the archival materials was removed and stored at –70°C.

### PCR-SSCP

The polymerase chain reaction single-stranded conformation polymorphism (PCR-SSCP) analysis was performed using a method previously described (Orita et al, 1989). Briefly, 80 ng of DNA was subjected to PCR amplification in a total volume of 30 µl of reaction mixture containing 10 mM Tris-HCl (pH 8.3), 50 mM potassium chloride, 1.5 mM magnesium chloride, 0.5 µM of each primer, 125 µM of each deoxyribonucleoside triphosphatase (dNTP), 0.8 µCi of [α-$^{32}$P]dCTP (specific activity 3000 Ci mmol$^{-1}$; Amersham, Bucks, UK), and 0.5 U of *Taq* Polymerase (Perkin Elmer, Roche Molecular Systems, Branchburg, NJ, USA). Thirty-five cycles of denaturation (95°C) for 50 s, annealing (58°C) for 50 s and extension (72°C) for 70 were carried out using an automated DNA thermal cycler (Omnigene thermal cycler, Hybaid, Teddington, UK). Four pairs of primers specific for exons 5–8 of the p53 gene were used:

exon 5: (5F) 5′ TTCCTCTTCCTGCAGTACTC 3′

(5R) 5′ CAGCTGCTCACCATCGCTAT 3′

exon 6: (6F) 5′ CACTGATTGCTCTTAGGTCT 3′

(6R) 5′ AGTTGCAAACCAGACCTCAG 3′

exon 7: (7F) 5′ TCTCCTAGGTTGGCTCTGAC 3′

(7R) 5′ CAAGTGGCTCCTGACCTGGA 3′

exon 8: (8F) 5′ CCTATCCTGAGTAGTGGTAATC 3′

(8R) 5′ CTTGCTTACCTCGCTTAGTG 3′

**Table 2** The relationship between immunohistochemical p53 nuclear staining and p53 mutation with a change in amino acid sequence or a stop codon

| | Immunohistochemical p53 staining | | | |
|---|---|---|---|---|
| | (<10%) | (10–70%) | (>70%) | *n* |
| p53 mutation | | | | |
| Yes | 4 | 4 | 4 | 12 |
| No | 29 | 15 | 21 | 65 |
| *n* | 33 | 19 | 25 | 77 |

**Table 3** Description of p53 gene mutations and variants found in 77 squamous cell carcinomas of the head and neck

| Case | Exon | Codon | Nucleotide | Amino acid | IHC |
|---|---|---|---|---|---|
| 126 | 5 | 141 | TGC → TGA | Cys to Stop | *** |
| 77 | 5 | 150 | ACA → ATA | Thr to Ile | ** |
| 125[a] | 5 | 177 | CCC → TCC | Pro to Ser | * |
| 204[a] | 6 | 192 | CAG → TAG | Gln to Stop | * |
| 60 | 6 | 216 | GTG → ATG | Val to Met | *** |
| 191 | 7 | 237 | ATG → ATC | Met to Ile | ** |
| 99 | 7 | 241 | TCC → TGC | Ser to Cys | * |
| 172 | 7 | 245 | GGC → AGC | Gly to Ser | *** |
| 193[a] | 7 | 245 | GGC → TGC | Gly to Cys | *** |
| 205 | 8 | 279 | GGG → TGG | Gly to Trp | ** |
| 56 | 8 | 281 | GAC → TAC | Asp to Tyr | ** |
| 86[a] | 8 | 290 | CGC → CTC | Arg to Leu | * |
| 186[b] | 5 | 142 | CCT → CCA | Pro to Pro | *** |
| 190[b] | 7 | 248 | CGG → AGG | Arg to Arg | ** |
| 85[b] | 7 | 249 | AGG → AGA | Arg to Arg | * |
| 73[b] | 8 | 282 | CGG → AGG | Arg to Arg | *** |
| 122[b] | 8 | 282 | CGG → AGG | Arg to Arg | * |

[a]Excluded from survival analysis because of lack of follow-up. [b]Silent variants excluded from survival analysis. IHC, immunohistochemical staining scores: * <10% nuclei stained; ** 10–70% nuclei stained; *** >70% nuclei stained.

For SSCP aliquots (1–5 µl) of the amplification mixture were mixed with a sequence stop solution (9–5 µl) (98% deionized formamide, 10 mM EDTA (pH 58.0), 0.025% xylencyanol and 0.025% bromophenol blue), heated at 95°C for 5 min and immediately loaded on to a 6% non-denaturing polyacrylamide gel containing 5% glycerol. Gels were run at 8 W for 13–14 h at room temperature, wrapped in thin plastic film and autoradiography was performed for 24–72 h.

### DNA sequencing

Positive samples were directly sequenced by the dideoxy chain termination method using the Sequenase Version 2.0 kit (United States Biochemical, Cleveland, OH, USA), following the isolation of single-stranded DNA by means of Dynabeads M-280 Streptavidin (Dynal, Oslo, Norway). Briefly, using oligonucleotide primers biotinylated at the 5′-end of the coding strand, PCR amplification was performed with 160 ng of genomic DNA. The conditions of amplification were the same as those for PCR-SSCP except for omitting α-$^{32}$PdCTP and using biotinylated primers. An aliquot of 25 µl of the PCR mixture was used to isolate single-stranded DNA, according to the manufacturer's

 © Cancer Research Campaign 1998

**Table 4** Distribution of mutation with respect to T stage. N stage and clinical stage in 58 HNSCCS evaluated for survival

| | p53 mutation ($n$ = 8) | No mutation ($n$ = 50) |
|---|---|---|
| T1 | 1 | 9 |
| T2 | 5 | 21 |
| T3 | 1 | 11 |
| T4 | 1 | 9 |
| N0 | 4 | 35 |
| N+ | 4 | 15 |
| Stage I–II | 3 | 21 |
| Stage III–IV | 5 | 29 |

directions. The samples were electrophoresed through 4.5% polyacrylamide gel containing 8.3 M urea for 1–2 h at 45 W, and the subsequently dried gel was exposed to KodakX-AR (Eastman Kodak Company, Rochester, NY) film for 48–72 h. Primers used were as follows:

exon 5: (5F) 5′ biotin-TTCAACTCTGTCTCCTTCCT 3′
(5R) 5′ GCAATCAGTGAGGAATCAGA 3′
sequencing primer:
5′ CAGCCCTGTCGTCTCCAG 3′
exon 6: (6F) biotinylated primer, the same as for exon 5
(6R) 5′ CGGAGGGCCACTGACAACCA 3′
sequencing primer:
5′ TTAACCCCTCCTCCCAGAGA 3′
exon 7: (7F) 5′ biotin-AGGCGCACTGGCCTCATCTT 3′
(7R) 5′ AGGGGTCAGCGGCAAGCAGA 3′
sequencing primer:
5′ TGTGCAGGGTGGCAAGTGGC 3′
exon 8: (8F) 5′ biotin-TTGGGAGTAGATGGAGCCT 3′
(8R) 5′ AGTGTTAGACTGGAAACTTT 3′
sequencing primer:
5′ AGGCATAACTGCACCCTTGG 3′

### Survival analysis

All medical records were reviewed for survival analysis. Patients with previous malignancies, recurrent tumours, treatment other than for cure or death within 3 months after diagnosis were excluded. This left 58 evaluable cases. Follow-up, performed on an ambulant basis after completed therapy, extended to 30 August, 1995. Patients were thus followed for at least 9 months or until death. Median duration of follow-up was 39.2 months (mean 45.3 months; range 3.6–90.7 months). Only six patients were followed less than 2 years.

**Figure 2** Kaplan–Meier survival curve with respect to cancer death only for the groups with and without mutations as determined by conformational changes of p53 ($n$ = 58; $P$ < 0.005)

### Statistical methods

Statistical analysis was performed with SPSS (Statistical Package for the Social Sciences) 6.1 (SPSS, Chicago, IL, USA). The Kaplan–Meier method was used for plotting survival curves. Log-rank test was used for survival analysis. Multivariate analysis (Cox's proportional hazards model) was used to test whether the differences were confounded by other host or tumour factors. Possible differences in the distribution of those factors between the different groups were investigated using the chi-square test, Fisher's exact test or Student's $t$-test. $P$-values quoted were two-tailed and were considered statistically significant when less than 0.05.

### RESULTS

The cancer samples generally had little stromal cell contamination and only a few samples contained a minor fraction of tumour cells. In general, two-thirds or more of the slides consisted of tumour cells. Only 3 out of the 77 biopsy specimens studied were considered to have scant tumour. The first had no detectable p53 mutation and high (>70%) nuclear staining. The second had no detectable p53 mutation, and less than 10% nuclear staining. The third had a p53 mutation and less than 10% nuclear staining. Only the second patient was eligible for survival analysis (treated for cure, no previous malignancy).

### Immunohistochemistry

Forty-four cases (57.1%) of the 77 analysed tumours revealed immunohistochemical positivity for p53 (≥ 10% staining nuclei).

**Table 5** Multivariate analysis of risk factors in 58 HNSCCs evaluated for cause-specific survival

| Variable | Relative risk | 95% confidence interval | Significance level |
|---|---|---|---|
| N0 vs N+ | 2.57 | 0.85–7.84 | 0.0960 |
| T-stage 1–4 | 1.72 | 0.50–5.95 | 0.3895 |
| Clinical stage I–IV | 1.20 | 0.28–5.25 | 0.8052 |
| Age | 0.74 | 0.29–1.85 | 0.5159 |
| p53 mutation | 9.87 | 3.21–30.34 | 0.0001 |

© Cancer Research Campaign 1998

**Figure 3** Kaplan–Meier survival curve with respect to cancer-only death for the groups with low (<10% staining nuclei), intermediate (10–70% staining nuclei) and high (>70% staining nuclei) p53 expression ($n$ = 58; $P$ = NS)

Among them 25 cases were scored 3 (more than 70% staining nuclei) and 19 cases were scored 2 (between 10% and 70% staining nuclei). Thirty-three cases (42.9%) showed score 1 (less than 10% staining nuclei) (Table 2).

### p53 mutations

Nucleotide sequence alterations were found in 17 (22%) of the 77 analysed tumours. Ten of these were missense mutations resulting in amino acid substitutions, two were nonsense mutations resulting in protein truncation, whereas five alterations would not lead to amino acid changes (Table 3). Thus, only 12 (16% of 77) tumours exhibited mutations that could alter the function of the protein. Seven of these were transversion mutations (four G → T, one C → A, one C → G, one G → G), and five were transitions (two G → A and three C → T, none at CpG dinucleotides). In the following computations, only cases exhibiting missense or nonsense mutation were included.

### PCR-SSCP vs IHC analysis

The concordance between p53 mutation, resulting in a missense or nonsense mutation, and increased p53 expression in immunohistochemistry was poor (Table 2). If only cases with either high or low IHC expression ($n$ = 58) are considered, IHC and PCR-SSCP analysis were discordant in 25 cases (43%). A SSCP gel, sequencing gel and immunohistochemistry staining of a tumour with concordant findings are shown in Figure 1.

Among 33 cases with no p53 expression immunohistochemically, four cases (12.1%) demonstrated mutations and 29 cases (87.9%) did not. Four (21.1%) out of 19 cases scoring 2 immunohistochemically had mutations, and four cases (16.0%) out of 25 cases scoring 3 had mutations. This result did not reveal any significant correlation between p53 mutation and p53 overexpression (Table 2).

### Survival vs p53 mutation/overexpression

Eight of 58 evaluable cases exhibited a p53 mutation resulting in a missense or nonsense mutation (Table 3). Of these 58 tumours, 48% exhibited low p53 IHC (<10% staining nuclei), 26% intermediate staining (10–70%) and 26% high staining (>70%). Neither for p53 mutation (missense, nonsense and silent) nor for p53 immunohistochemical overexpression (data not shown) were there any differences in the distribution of T or N status, stage, age or sex between the groups (Table 4).

Univariate analysis revealed both N status ($P$ = 0.01, log rank) and p53 mutation ($P$ = 0.001, log rank) (Figure 2) to be associated with survival. This was, however, not the case for immunohistochemical expression of p53 (Figure 3). Median survival for patients with missense or nonsense p53 mutation was 12.5 months and for patients without such mutations median survival extended beyond 90 months. In a further subgroup, survival analysis of p53-mutated and non-mutated cases was subdivided into groups with or without increased immunohistochemical p53 expression. Whether or not the cut-off limit was between <10% and > 10% staining nuclei, or between <70% and >70% staining nuclei (data not shown), no further prognostic information was gained. Using this subdivision some of the groups, however, were small.

In a multivariate analysis, p53 mutation was still a strong risk factor and the impact of N status was reduced below significance (Table 5).

### DISCUSSION

p53 mutations that lead to altered protein conformation can make the protein more stable and prolong its half-life (Lane and Benchimol, 1990). It is therefore possible to detect an accumulation of mutated p53 protein in head and neck cancer using immunohistochemistry. The frequency of p53 immunohistochemical overexpression in the present material (57% with > 10% staining nuclei) is in accordance with findings in previous studies of HNSCC (Field et al, 1991, 1993; Ogden et al, 1992; Watling et al, 1992; Dowell and Hall, 1994; Xu et al, 1994; Nylander et al, 1995).

As we previously reported in preliminary form (Mineta et al, 1995), there is a pronounced discordance between p53 mutation and p53 immunohistochemical overexpression. This confirms some earlier findings in HNSCC (Xu et al, 1994; Nylander et al, 1995) and in skin cancers (Kubo et al, 1994), but contradicts others (Ahomadegbe et al, 1995). The difference in findings is not likely to be attributed to the antibody used as DO-7 was applied in the present as well as in two (Ahomadegbe et al, 1995; Nylander et al, 1995) of the four other studies.

False-negative findings (mutation without overexpression) can be attributed to p53 mutations at splice sites, frame shifts or nonsense mutations, which would be predicted to encode for truncated p53 proteins not detected by immmunohistochemistry. Studies of the crystal structure of the p53 tumour suppressor–DNA complex (Cho et al, 1994; Milner, 1995) have made it clear that the majority of p53 point mutations affect the residues within the core domain and inactivate the function by abolishing its sequence-specific DNA-binding capacity. However, these mutations do not significantly affect the structure of the protein.

Therefore, the discrepancy between the PCR-SSCP and the immunohistochemical results may depend on the site of p53 mutation or the anti-p53 antibody employed. Negative staining may also be due to overfixation, delayed fixation or inadequate tissue processing of the tumour sample, which allows degradation of the p53 protein.

The p53 antibody (Do-7) used in this study reacts with both the wild type and mutant type of the p53 protein. Our data, which

© Cancer Research Campaign 1998

demonstrate three cases with p53 missense mutations not detected immunohistochemically, suggest that the mutational change in the p53 gene does not necessarily result in increased stability of the p53 protein.

Discordant results (i.e. nuclear staining without p53 mutation) can depend on the sequencing strategy. For example, mutations may occur outside the studied exons 5, 6, 7 and 8. For HNSCC, however, approximately 98% of mutations are found within exons 5–8 (Greenblatt et al, 1994). Other reasons for the failure of detection of p53 mutations include insufficient tumour in the sample or insufficient quality of DNA from archival materials.

Overexpression is not only seen in gene mutation, but also in cases with retention of the wild-type target protein in the tumour cell. Both mutational stabilization of the p53 protein and elevated levels of wild-type p53 protein allow detection by immunohistochemistry. Thus, if the secondary stabilization of p53 occurs by some mechanism other than gene mutation, overexpression can be demonstrated.

Accumulation of wild-type p53 protein has been found in an inherited cancer (Barnes et al, 1992) or cancer treated with chemotherapeutic drugs or radiation (Kastan et al, 1991; Vogelstein and Kinzler, 1992).

Such non-mutational stabilization of the protein is most probably the result of interruption of the normal degradative pathway of p53. Other proteins such as the products of cellular oncogene *mdm-2* (Monaud et al, 1992; Meltzer, 1994), or the products of DNA viruses, including SV-40 large T antigen, E1b of adenovirus (Gannon et al, 1990; Cesarman et al, 1993) and E6 of HPV (Scheffner et al, 1990; Werness et al, 1990) can bind to the p53 gene and inactivate the ability to act as a transcription factor, resulting in p53 stabilization. Phosphorylation due to cdc2-like kinase could also alter the p53 protein, which can then be detected by immunohistochemistry (Moll et al, 1992).

In the present study p53 mutations were found at a frequency of 16% (12/77), which is within the lower range of the percentage of abnormal findings reported in the literature in HNSCC (Boyle et al, 1993; Brennan et al, 1995; Dowell and Hall, 1994; Greenblatt et al, 1994; Xu et al, 1994; Nylander et al, 1995). The low frequency may reflect the small sample size currently available, or it may be due to inconsistent amplification of DNA from archival materials, or to lower mutated DNA concentration in samples, although mutations are reported to be still detectable when constituting only 15% of total DNA (Wu and Darras, 1993).

The issue of decreased sensitivity caused by contaminating benign cells is of greatest importance in PCR studies involving detection of loss of heterozygosity, for example. In studies such as ours, it is unclear how much contaminating cells lower the threshold of detection of p53 mutation in formalin-fixed material, if at all, and a sample judged insufficient in the amount of tumour for immunohistochemical assessment produced evidence of p53 mutation in the PCR. The proportion of tumour cells in all samples vastly exceeded, in any case, the generally accepted sensitivity of PCR in detecting 1 mutated cell among 100 000 normal cells.

The simple possibility that a dissimilarity exists between cohorts of patients with respect to carcinogen exposure may influence the findings. It also appears as though the frequency of detected mutations may be lower when DNA is retrieved from archival rather than from fresh tissue.

Studies have implicated p53 protein expression as an independent prognostic factor in carcinomas of the breast, stomach, colon/rectum, bladder and NSCLC [reviewed in Chang et al (1995), Dowell and Hall (1994)]. The clinical relevance of p53 overexpression in HNSCC has been under debate. We could not find any correlation between p53 immunohistochemical overexpression and survival (Figure 3). There are studies indicating a correlation between p53 overexpression and survival, some reporting better survival in patients with overexpression (Sauter et al, 1992). Overexpression has also been reported to show strong association with a histological malignancy grading scale with prognostic capability (Watling et al, 1992). However, the lack of correlation between p53 expression and clinicopathological parameters or survival as originally reported by Field et al (1991) has subsequently been substantiated by many reports [reviewed in Field et al (1993)].

Studies on the relation between p53 mutation in HNSCC and clinicopathological parameters or survival are sparse. Koch et al (1996) found an association with recurrence but not survival. The present finding of p53 mutation as a strong and independent prognostic variable contrasts with the results of Ahomadegbe et al (1995), who did not find any correlation between mutation and clinical stage or 5-year survival. However, they studied fresh tissue from both metastases ($n = 50$) and primary tumours ($n = 28$), 13 of which were matched specimens. They also found a good correlation between mutation and overexpression. Nylander et al (1995) in a study of 80 HNSCCs of the oral cavity using archival specimens could not find any relation between p53 mutation and survival. Their material, however, deviated from the general characteristics of HNSCC, with a male to female ratio of 0.86:1 and a high frequency of a novel non-random 14-bp deletion in exon 8 (Nylander et al, 1996), differences that might explain the discordant findings with respect to survival.

In conclusion, we verified previous findings of a lack of concordance between immunohistochemical overexpression of nuclear p53 and mutation of the p53 gene, as well as the absence of prognostic information with respect to survival in p53 overexpression. On the other hand, p53 mutation seems to be a strong and independent variable for survival prognosis.

### ACKNOWLEDGEMENTS

This investigation was supported by grants from the Swedish Cancer Society (1304-B95–09XAC, 1304-B96–10XAA, 1304-B95–08PBC), King Gustaf V's Jubilee Fund (96:529), the Foundations of Lund's Health District Organisation and the Research Funds of the Medical Faculty of the University of Lund, Sweden.

### REFERENCES

Ahomadegbe JC, Barrois M, Fogel S, Le Bihan ML, Douc-Rasy S, Duvillard P, Armand JP and Riou G (1995) High incidence of p53 alterations (mutation, deletion, overexpression) in head and neck primary tumors and metastases: absence of correlation with clinical outcome. Frequent protein overexpression in normal epithelium and in early non-invasive lesions. *Oncogene* **10**: 1217–1227

Barnes DM, Handy AM, Gillett CE, Mohammed S, Hodgson S, Bobrow LG, Leigh IM, Purkis T, MacGeoch C, Spurr NK, Bartek J, Vojtesek B, Picksley SM and Lane DP (1992) Abnormal expression of wild type p53 protein in normal cells of a cancer family patient. *Lancet* **340**: 259–263

Boyle J, Hakim J, Koch W, van der Riet P, Hruban RH, Roa RA, Correo R, Eby YJ, Ruppert JM and Sidransky D (1993) The incidence of p53 mutations increases with progression of head and neck cancer. *Cancer Res* **53**: 4477–4480

Brennan JA, Boyle JO, Koch WM, Goodman SN, Hruban RH, Eby YJ, Couch MJ, Forastiere AA and Sidransky D (1995) Association between cigarette smoking

© Cancer Research Campaign 1998

and mutation of the p53 gene in squamous-cell carcinoma of the head and neck. *N Engl J Med* **332**: 712–717

Cesarman E. Inghirami G. Chadburn A and Knowles DM (1993) High levels of p53 protein expression do not correlate with p53 gene mutations in anaplastic large cell lymphoma. *Am J Pathol* **143**: 845–856

Chang F. Syrjänen S and Syrjänen K (1995) Implications of the p53 tumor-suppressor gene in clinical oncology. *J Clin Oncol* **13**: 1009–1022

Cho Y. Gorina S. Jeffrey PD and Pavletich NP (1994) Crystal structure of a p53 tumor suppressor–DNA complex: understanding tumorigenic mutations. *Science* **265**: 346–355

Dowell SP and Hall PA (1994) The clinical relevance of the p53 tumour suppressor gene. *Cytopathology* **5**: 133–145

el-Naggar AK. Lai S. Luna MA. Zhou XD. Weber RS. Goepfert H and Batsakis JG (1995) Sequential p53 mutation analysis of pre-invasive and invasive head and neck squamous carcinoma. *Int J Cancer* **64**: 196–201

Field JK. Spandidos DA. Malliri A. Gosney JR. Yiagnisis M and Stell PM (1991) Elevated p53 expression correlates with a history if heavy smoking in squamous cell carcinoma of the head and neck. *Br J Cancer* **64**: 573–577

Field JK. Spandidos DA and Stell PM (1992) Over-expression of p53 gene in head and neck cancer. linked with heavy smoking and drinking. *Lancet* **339**: 502–503

Field JK. Pavelic ZP. Spandidos DA. Stambrook PJ. Jones AS and Gluckman JL (1993) The role of the p53 tumor suppressor gene in squamous cell carcinoma of the head and neck. *Arch Otolaryngol Head Neck Surg* **119**: 1118–1122

Gannon JV. Greaves R. Iggo R and Lane DP (1990) Activating mutations in p53 produce a common conformational effect. A monoclonal antibody specific for the mutant form. *EMBO J* **9**: 1595–1602

Greenblatt MS. Bennett WP. Hollstein M and Harris CC (1994) Mutations in the p53 tumour suppressor gene: clues to cancer etiology and molecular pathogenesis. *Cancer Res* **54**: 4855–4878

Harris CC and Hollstein M (1993) Clinical implications of the p53 tumor-suppressor gene. *N Engl J Med* **329**: 1318–1327

Hermanek P and Sobin LH (1987) TNM classification of malignant tumours. pp. 13–33. Springer-Verlag: Berlin

Kastan MB. Onyinye O. Sidransky D. Vogelstein B and Craig RW (1991) Participation of p53 protein in the cellular response to DNA damage. *Cancer Res* **51**: 6304–6311

Koch WM. Brennan JA. Zahurak M. Goodman SN. Westra WH. Schwab D. Yoo GH. Lee DJ. Forastiere AA and Sidransky D (1996) p53 mutation and locoregional treatment failure in head and neck squamous cell carcinoma. *J Natl Cancer Inst* **88**: 1580–1586

Kubo Y. Urano Y. Yoshimoto K. Iwahana H. Fukuhara K. Arase S and Itakura M (1994) p53 gene mutations in human skin cancers and precancerous lesions: comparison with immunohistochemical analysis. *J Invest Dermatol* **102**: 440–444

Landrigan PJ and Baker DB (1991) The recognition and control of occupational disease. *JAMA* **266**: 676–680

Lane DP and Benchimol S (1990) p53: oncogene or anti-oncogene? *Genes Dev* **4**: 1–8

Levine AJ. Momand J and Finlay CA (1991) The p53 tumour suppressor gene. *Nature* **351**: 453–456

Levine AJ. Perry ME. Chang A. Silver A. Dittmer D. Wu M and Welsh D (1994) The 1993 Walter Hubert Lecture: the role of the p53 tumour-suppressor gene in tumorigenesis. *Br J Cancer* **69**: 409–416

Lungu O. Wright TCj and Silverstein S (1992) Typing of human papillomaviruses by polymerase chain reaction amplification with L1 consensus primers and RFLP analysis. *Mol Cell Probes* **6**: 145–152

Meltzer PS (1994) MDM2 and p53: a question of balance. *J Natl Cancer Inst* **86**: 1265–1266

Milner J (1995) DNA damage. p53 and anticancer therapies. *Nature Med* **1**: 879–80

Mineta H. Borg Å. Dictor M. Wahlberg P and Wennerberg J (1995) Discordance between p53 protein expression and suppressor gene mutation in H&N squamous cell carcinoma. *Eur J Cancer* **31A** (suppl. 5): 92

Moll UM. Rou. G and Levine AJ (1992) Two distinct mechanisms alter p53 in breast cancer: mutation and nuclear exclusion. *Proc Natl Acad USA* **89**: 7262–7266

Monaud J. Zambetti GP. Olson DC. George D and Levine AJ (1992) The mdm-2 oncogene product forms a complex with the p53 protein and inhibits p53-mediated transactivation. *Cell* **69**: 1237–1245

Nees M. Homann N. Discher H. Andl T. Enders C. Herold-Mende C. Schumann A and Bosch FX (1993) Expression of mutated p53 occurs in tumor-distant epithelia of head and neck cancer patients: a possible molecular basis for the development of multiple tumors. *Cancer Res* **53**: 4189–4196

Nylander K (1995) Squamous cell carcinoma of the head and neck – proliferation. p53 and prognosis. In *Dept of Oral Pathology and Pathology* pp. 91. Umeå University: Umeå

Nylander K. Nilsson P. Mehle C and Roos G (1995) p53 mutations. protein expression and cell proliferation in squamous cell carcinomas of the head and neck. *Br J Cancer* **71**: 826–830

Nylander K. Schildt EB. Eriksson M. Magnusson A. Mehle C and Roos G (1996) A non-random deletion in the p53 gene in oral squamous cell carcinoma. *Br J Cancer* **73**: 1381–1386

Ogden GR. Kiddie RA. Lunny DP and Lane DP (1992) Assessment of p53 protein expression in normal. benign and malignant oral mucosa. *J Pathol* **166**: 389–394

Orita M. Iwahana H. Kanazawa H. Hayashi K and Sekiya T (1989) Detection of polymorphisms of human DNA by gel electrophoresis as single-strand conformation polymorphisms. *Proc Natl Acad Sci* **86**: 2766–2770

Pavelic ZP. Li YQ. Stambrook PJ. McDonalds JS. Munck-Wikland E. Pavelic K. Dacic S. Danilovic Z. Pavelic L. Mugge RE.Wilson K. Nguyen C and Gluckman JL (1994) Overexpression of p53 protein is common in premalignant head and neck lesions. *Anticancer Res* **14**: 2259–2266

Sauter ER. Ridge JA. Gordon J and Eisenberg BL (1992) p53 overexpression correlates with increased survival in patients with squamous carcinoma of the tongue base. *Am J Surg* **164**: 651–653

Scheffner M. Werness BA. Hibregtse JM. Levine AJ and Howley PM (1990) The E6 oncoprotein encoded by human papillomavirus types 16 and 18 promotes the degradation of p53. *Cell* **63**: 1129–1136

Shin DM. Kim J. Ro JY. Hittelman J. Roth JA. Hong WK and Hittelman WN (1994) Activation of p53 gene expression in premalignant lesions during head and neck tumorigenesis. *Cancer Res* **54**: 321–326

Vogelstein N and Kinzler KW (1992) p53 function and dysfunction. *Cell* **70**: 523–526

Wu JK and Darras BT (1993) Sensitivity of single-strand conformation polymorphism (SSCP) analysis in detecting p53 point mutations in tumours with mixed cell populations. *Am J Hum Genet* **52**: 1273–1275

Wang LD. Shi ST. Zhou Q. Goldstein S. Hong JY. Shao P. Qiu SL and Yang CS (1994) Changes in p53 and cyclin D1 protein levels and cell proliferation in different stages of human oesophageal and gastric-cardia carcinogenesis. *Int J Cancer* **59**: 514–519

Watling DL. Gown AM and Coltrera MD (1992) Overexpression of p53 in head and neck cancer. *Head and Neck* **14**: 437–444

Warness BA. Levine AJ and Howley PM (1990) Association of human papillomavirus types 16 and 18 E6 proteins with p53. *Science* **248**: 76–79

Xu L. Chen YT. Huvos AG. Zlotolow IM. Rettig WJ. Old. LJ & P. G-C (1994) Overexpression of p53 protein in squamous cell carcinomas of the head and neck without apparent gene mutations. *Diagn Mol Pathol* **3**: 83–92

Zätterström UK. Wennerberg J. Ewers S-B. Willén R and Attewell R (1991) Prognostic factors in head and neck cancer: histologic grading. DNA ploidy and nodal status. *Head Neck* **13**: 477–487

© Cancer Research Campaign 1998